# Draft Genome Sequence of a Potentially Novel Streptococcus Species Belonging to the Streptococcus mitis Group

**DOI:** 10.1128/genomeA.00620-18

**Published:** 2018-06-28

**Authors:** Ingerid Ø. Kirkeleite, Jon Bohlin, Lonneke Scheffer, Einar T. Weme, Didrik F. Vestrheim

**Affiliations:** a Division of Infection Control and Environmental Sciences, Norwegian Institute of Public Health, Oslo, Norway; b Department of Informatics, University of Oslo, Oslo, Norway; c Department of Medical Microbiology, Vestre Viken Hospital Trust, Drammen, Norway

## Abstract

We report here the draft genome sequence of a Streptococcus species belonging to the S. mitis group. While a clear species identification cannot be made for the isolate, it appears that its most recent common ancestor is the species S. pseudopneumoniae.

## GENOME ANNOUNCEMENT

The mitis group of streptococci comprises 17 known species of Gram-positive bacteria, including Streptococcus pneumoniae, S. mitis, and S. pseudopneumoniae, according to its latest taxonomy (1). Members of the mitis group are found among the commensal flora of the oral cavity and upper respiratory tract. S. pneumoniae is a frequent cause of respiratory tract infections, such as pneumonia, as well as invasive diseases like meningitis and septicemia (2–4).

Most members of the mitis group are naturally susceptible to genetic transformation by homologous recombination and exhibit substantial intraspecies variations, both phenotypically and genetically (5–8). Due to phenotypic overlap and genetic heterogeneity, it has been challenging to determine the species of commensal members of the mitis group, especially using 16S rRNA-based sequence analysis (1, 9). Indeed, the most notable difference among the members of the S. mitis group is their pathogenic potential (9). Recent studies suggest that the commensal strains of the mitis lineage may have evolved from a pathogenic ancestor through the loss of virulence genes and reductive evolution (8, 9). This is supported by the reduced genome sizes of these commensals.

Here, we present the draft genome of a potentially novel Streptococcus species that seems to have evolved from a common pathogenic ancestor of S. pneumoniae and S. pseudopneumoniae, with the latter being putatively its most recent common ancestor.

The bacterium in question, designated isolate 596553, was recovered from a nasopharyngeal swab specimen obtained from a patient with coronary artery disease, alcoholic encephalopathy, and possibly myelofibrosis. The patient was suffering from high fever and lung congestion and had low hemoglobin levels before receiving a blood transfusion. The strain was determined to belong to the mitis group of the genus Streptococcus by conventional phenotypical and biochemical measures, such as optochin disc susceptibility, bile solubility, and matrix-assisted laser desorption ionization–time of flight mass spectrometry (MALDI-TOF) Quellung reaction (not typeable and negative reaction with omniserum). Phenotypic species-level identification did not, however, give conclusive results. To facilitate a more thorough species identification of isolate 596553, we employed whole-genome sequencing technology.

We isolated genomic DNA using the MagNA Pure 96 system and sequenced the genome with the Illumina MiSeq platform. We then *de novo* assembled the genome using SPAdes version 3.7 (10). Using SpeciesFinder (https://cge.cbs.dtu.dk/services/SpeciesFinder), we identified the isolate to be similar to S. mitis. The 16S rRNA analysis was inconclusive but suggested S. pneumoniae and S. pseudopneumoniae as the most likely candidates. Single-nucleotide polymorphism analysis (11) was subsequently performed on the following group of streptococci: S. mitis B6 (accession number NC_013853), S. pneumoniae SPN032672 (accession number NC_021003), and S. pseudopneumoniae IS7493 (accession number CP002925), all closed genomes available at GenBank (12), together with isolate 596553. Isolate 596553 did not, however, cluster with the other streptococci. Finally, a protein-by-protein comparison (13, 14) suggested that the closest species was S. pseudopneumoniae IS7493, with approximately 81% protein similarity. Hence, we cannot determine which species is appropriate for isolate 596553, but it is clear that it belongs to the mitis group.

### Accession number(s).

The draft genome sequence of S. mitis isolate 596553 has been deposited in GenBank under the accession number OXCQ00000000. Isolate 596553 is available for further analysis at ENA (EMBL-EBI) under the study accession number PRJEB26772.
